# Asymmetric coupling of action and outcome valence in active and observational feedback learning

**DOI:** 10.1007/s00426-020-01340-1

**Published:** 2020-04-22

**Authors:** Jutta Peterburs, Alena Frieling, Christian Bellebaum

**Affiliations:** Department of Biological Psychology, Institute of Experimental Psychology, Heinrich-Heine-University Düsseldorf, Universitätsstraße 1, 40225 Düsseldorf, Germany

## Abstract

Learning to execute a response to obtain a reward or to inhibit a response to avoid punishment is much easier than learning the reverse, which has been referred to as “Pavlovian” biases. Despite a growing body of research into similarities and differences between active and observational learning, it is as yet unclear if Pavlovian learning biases are specific for active task performance, i.e., learning from feedback provided for one’s own actions, or if they persist also when learning by observing another person’s actions and subsequent outcomes. The present study, therefore, investigated the influence of action and outcome valence in active and observational feedback learning. Healthy adult volunteers completed a go/nogo task that decoupled outcome valence (win/loss) and action (execution/inhibition) either actively or by observing a virtual co-player’s responses and subsequent feedback. Moreover, in a more naturalistic follow-up experiment, pairs of subjects were tested with the same task, with one subject as active learner and the other as observational learner. The results revealed Pavlovian learning biases both in active and in observational learning, with learning of go responses facilitated in the context of reward obtainment, and learning of nogo responses facilitated in the context of loss avoidance. Although the neural correlates of active and observational feedback learning have been shown to differ to some extent, these findings suggest similar mechanisms to underlie both types of learning with respect to the influence of Pavlovian biases. Moreover, performance levels and result patterns were similar in those observational learners who had observed a virtual co-player and those who had completed the task together with an active learner, suggesting that inclusion of a virtual co-player in a computerized task provides an effective manipulation of agency.

**Electronic supplementary material:**

The online version of this article (10.1007/s00426-020-01340-1) contains supplementary material, which is available to authorized users.

## Introduction

The ability to adjust behavior based on action consequences is critical in dynamic or novel environments. For instance, the familiar phrase “once bitten, twice shy” refers to the reluctance to repeat an action that has previously led to an unpleasant experience. The Law of Effect put forward by Edward Thorndike states that responses which produce a satisfying or pleasing effect in a particular situation become more likely to occur again in that situation, and responses that produce a discomforting effect or fail to elicit pleasure become less likely to occur again in that situation (Thorndike, 1927). But not all contingencies between actions (or decisions) and their outcomes are learned equally! Guitart-Masip et al. (2011) devised a task that decoupled action and outcome valence, the orthogonalized go/nogo task. Adding to previous evidence for a particular coupling between reward and go responses and between punishment and no-go responses (Gray & MacNaughton, 2003), they found that in this task, learning to execute a response to obtain a reward (go to win) or to inhibit a response to avoid punishment (nogo to avoid losing) was easier than learning the reverse (Guitart-Masip, Huys et al., 2012a, b), which has been referred to as “Pavlovian” biases. Interestingly, learning success depended on concerted recruitment of bilateral inferior frontal cortex in addition to midbrain regions belonging to the “reward system”, possibly indicating that brain regions implicated in response inhibition are needed to overcome Pavlovian control (Guitart-Masip, Huys et al., 2012a, b). In line with this, midfrontal theta power as an electrophysiological index of prefrontal control has been directly linked to the ability to overcome Pavlovian biases (Cavanagh, Eisenberg, Guitart-Masip, Huys, & Frank, 2013)

More recent research has highlighted the importance of the specific context in which learning occurs. Millner et al. (2018) showed that an aversive context can facilitate action depending on whether the aversive stimulus is present or impending. In their study, Pavlovian processes interfered with feedback-based learning by promoting action to escape when an ongoing aversive auditory stimulus was present, and by promoting behavioral inhibition (i.e., withholding of responses) when the same aversive stimulus could be avoided.

Findings on the role of the neurotransmitters dopamine for learning and representing action–outcome contingencies further corroborate the notion that the factors action and outcome valence interact. Results of a functional imaging study in subjects highly trained in the orthogonalized go/nogo task suggested that levodopa enhanced striatal and substantia nigra/ventral tegmental representations of actions associated with obtaining a reward, while neither representations of actions associated with avoiding punishment nor neural responses to reward as such were enhanced, thus underlining the role of dopamine for appetitively motivated behavior (Guitart-Masip, Chowdhury et al., 2012a, b).

For learning to occur it is not necessary to perform an action and bear the consequences oneself. In everyday life, we often observe other individuals’ actions and ensuing consequences. For instance, we may watch someone operate a ticket machine at a train station and obtain a ticket before repeating the observed actions ourselves to buy our own tickets. On the other hand, if we observed that a ticket was not obtained or that the machine returned too little change due to a malfunction or inappropriate use, we would not be likely to act in the same way. This simple example illustrates that Thorndike’s Law of Effect applies to both active and observational learning. However, previous findings are somewhat inconsistent with regard to whether the two learning types are similarly effective, and it is as yet unclear if the above described Pavlovian learning biases also apply to observational learning. Bellebaum et al. (e.g., 2010, 2012) reported that learning from positive or negative feedback in a probabilistic learning task was similarly effective in an active and observational context. In contrast, Nicolle, Symmonds, and Dolan (2011) found observational learning to be associated with impaired accuracy when choosing between two low-value options, which was related to (subjective) over-estimation of the likelihood of winning in case of the lowest-value stimulus, i.e., an optimistic bias. Aside from learning, decision making has also been reported to differ between active subjects and observers. While both groups made risky choices beyond pure rationality, actors were riskier than observers (Fernandez-Duque & Wifall, 2007).

It has been proposed that active and observational learning may differ in attentional allocation during learning (Cohn et al., 1994), or in the nature of the involved knowledge representation, with observational learning possibly requiring more explicit, declarative representations, and active learning relying more on procedural and non-declarative representations (Kelly et al., 2003). Related to the latter, another key difference may lie within a reduced necessity to integrate own actions and outcome-related information in the observational context. This notion is supported by electrophysiological studies showing that variations of agency (here: own vs. observed choices) modulate aspects of action outcome processing that are related to the integration of action and outcome information. For example, the magnitude of the feedback-related negativity (FRN), an event-related potential component that has been related to outcome processing and coding of reward prediction errors (Gehring & Willoughby, 2002; Holroyd & Coles, 2002; Miltner et al., 1997; Nieuwenhuis et al., 2004), has been shown to be reduced in observational compared to active learning (Bellebaum et al., 2010; Bellebaum & Colosio, 2014; Fukushima & Hiraki, 2009; Koban et al., 2012; Kobza et al., 2011; Yu & Zhou, 2006).

In general, previous research, therefore, points to at least partially distinct mechanisms underlying active and observational learning from feedback. This notion is corroborated by clinical studies in patients with Parkinson’s Disease (PD) in whom degeneration of dopaminergic neurons in the substantia nigra results in reduced dopaminergic input to the striatum (Kish et al., 1988). In the OFF medication stage, these patients exhibit a bias towards learning from negative feedback, likely due to facilitated disinhibition of striatal “nogo” neurons in response to negative feedback which then hampers action selection in the frontal cortex (Frank et al., 2004; Frank, 2005). Interestingly, this bias was not found for learning by observation, suggesting that dopaminergic input to the striatum may play a less prominent role in observational than in active learning (Kobza et al., 2012). It has been proposed that the integration of information about (own) actions and their outcomes takes place in the dorsal striatum, where prediction errors have been shown to be more strongly represented in active than observational learning (Bellebaum et al., 2012) and in instrumental than in classical conditioning (O'Doherty et al., 2004; Valentin & O'Doherty, 2009).

If active and observational learning indeed differ with respect to striatal involvement and the coding of action–outcome contingencies, it is conceivable that the asymmetric coupling of action and outcome valence is attenuated in observational learning. In line with this, recent findings suggest that ventral striatal involvement in processing monetary feedback gradually decreases from own actions, a friend’s actions, to a stranger’s actions (Morelli et al., 2018). On the other hand, the dorsal striatum has been suggested to play a key role in linking instrumental actions and outcomes during both active and observational learning (Cooper et al., 2012), which might entail a similar Pavlovian bias in active and observational learning.

The present study was aimed to clarify in a series of behavioral experiments with an orthogonalized go/nogo task if action and outcome valence also interact in observational outcome-based learning, and, if so, whether the Pavlovian bias is similarly pronounced in active and observational learning. In Experiment 1, one group of healthy adult subjects completed the task as active learners, while participants in a second group were observational learners yoked to the active subjects. Importantly, the task was fully computerized so that for observers, the active subject’s responses were presented on the computer screen and marked by a picture of a hand. Experiment 2 was conducted to see to what extent the observers’ performance depended on the response pattern of the observed subject and thus possibly reflected mere imitation of the responses they had watched. To this end, healthy adults observed a virtual active learner’s chance performance in the orthogonalized go/nogo task. Experiment 3, which entailed two groups of subjects again, active and observational learners, used a more naturalistic setting: pairs of subjects completed the task simultaneously, with one subject as active and the other as observational learner. In general, it was hypothesized that a Pavlovian bias would also occur in observational learning, with enhanced learning of go (vs. nogo) to win and nogo (vs. go) to avoid associations. Consistent with reduced striatal involvement in observational learning, however, the coupling of action and outcome valence was expected to be less strong in observational as compared to active learning.

## Experiment 1

### Subjects

Forty adult volunteers (33 females, 7 males) were recruited for participation at Heinrich-Heine-University Düsseldorf, Germany, by public advertisement and/or on social media. All had normal or corrected-to-normal vision. Mean age was 22.7 years (SD = 3.8; age range 18–37 years). None of the subjects had any history of neurological or psychiatric illnesses or was currently treated with neurotropic medication. All subjects were naïve to the study’s intent. IQ estimates were obtained with a multiple choice vocabulary test (Mehrfachwahl-Wortschatz-Intelligenztest B, MWT-B Lehrl et al., 1995), a German test to measure crystallized intelligence in which subjects are presented with 37 items in each of which one real German word has to be correctly identified among 4 non-words. Points are awarded for each correct answer, and total test scores are translated into IQ estimates by means of norm tables. IQ estimates obtained with the MWT-B have been shown to correlate reasonably well with global IQ scores (Lehrl et al., 1995). Mean IQ was 113.57 (SD = 10.25) in the present sample. Written informed consent was obtained from all participants prior to participation. Subjects received course credit for participation. The study conforms to the Declaration of Helsinki and received ethical clearance by the Ethics Board of the Faculty of Mathematics and Natural Sciences at Heinrich-Heine-University Düsseldorf, Germany.

### Experimental task

The experimental task was a variant of a go/nogo task specifically designed to decouple outcome valence and action (Guitart-Masip et al., 2011). In this game-like task, participants can choose between different behavioral options in order to receive or avoid losing points. Four combinations of action and outcome valence were balanced throughout the task: *go to win* points, *go to avoid losing* points, *nogo to win* points, and *nogo to avoid losing* points. Four abstract fractal images (Mathôt et al., 2015; obtained from https://github.com/smathot/materials_for_P0010.5) were used as imperative stimuli and randomly assigned to these combinations at the beginning of each test session. Separate subsamples of *N* = 20 subjects completed the task as active learners or observational learners, with each observational learner yoked to one actively learning subject. In order to allow for a comparable assessment of learning performance in both active and observational learners, the task comprised not only four (active or observational) learning blocks with feedback, but also four test blocks without feedback which required active responding by both active and observer participants. The types of blocks alternated, beginning with a learning block. Individual learning performance for both groups of participants was assessed based on test block performance (see below).

**Fig. 1:**
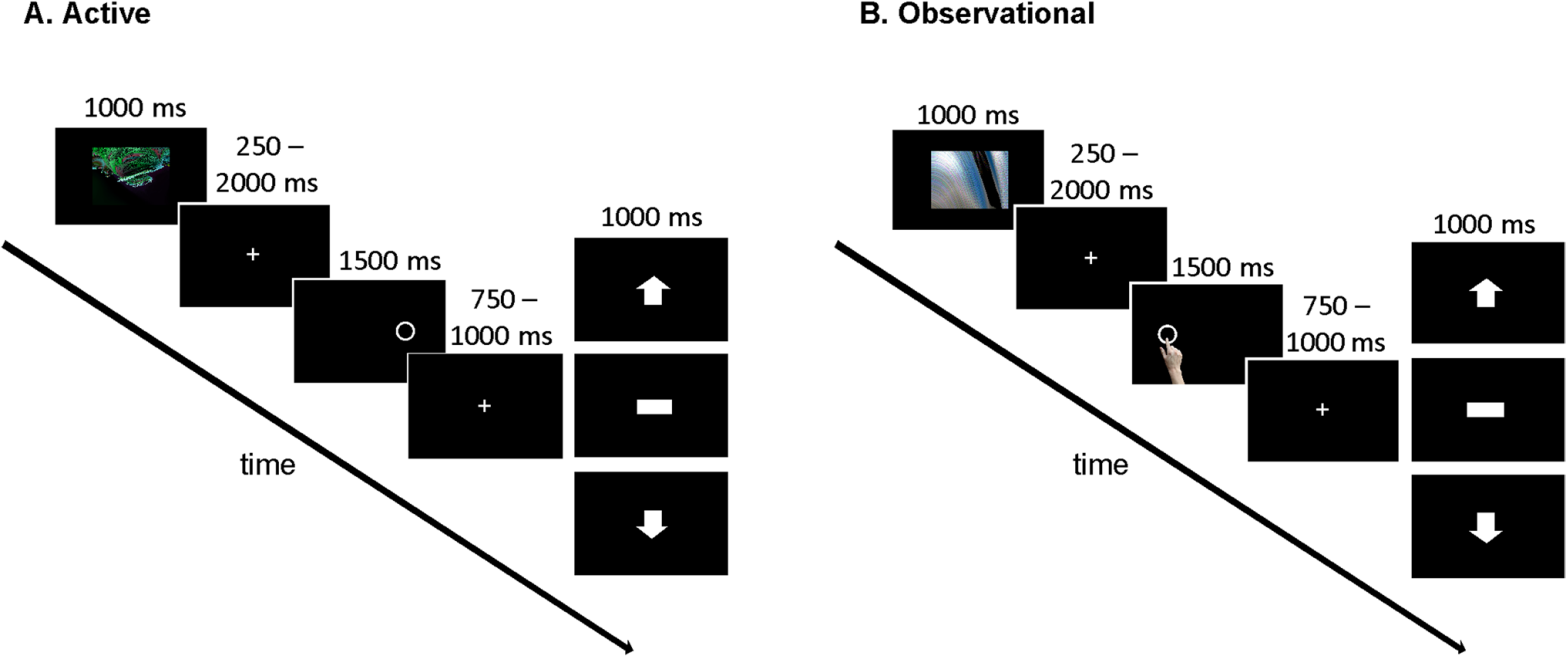
Schematic illustration of the sequence and time course of stimulus presentation in a single learning block trial in the active (**a**) and observational versions (**b**) of the go/nogo task. This task was specifically designed to decouple outcome valence (win/loss) and action (go/nogo)

Figure 1 illustrates the time course and sequence of stimulus presentation in trials in the learning block. In the task version for active learners (Fig. 1a), each trial started with a fractal image which was presented for 1000 ms, followed by a fixation cross for 250–2000 ms. Afterwards, an open circle was presented on the left or right side of the screen for 1500 ms. Subjects were instructed to decide between responding and not responding, and in case of responding to press the response button (left or right STRG key on a standard USB keyboard) corresponding to the side the circle had been presented on (e.g., left button for circle on left side). Responses were required to occur within 1000 ms of stimulus onset. In case the participants chose not to respond they had to let the response period pass. If they accidentally pressed the wrong button on the opposite side of the circle, they were explicitly informed about this and the trial was aborted. Following presentation of the circle cue, a fixation cross was displayed for 750–1000 ms, before symbolic feedback about the choice (response/no response) was provided. An upward pointing arrow indicated that 10 points had been gained (win), a downward pointing arrow indicated that 10 points had been lost (loss), and a horizontal bar indicated that no points had been gained or lost (draw). Throughout the task participants could learn which fractal stimulus was associated with which kind of outcome (win/draw/loss) for which kind of choice (go or nogo). For two stimuli, the “good” outcome was to avoid losing points (draw) and the alternative was a loss of points. For two others, a win was the favorable outcome and a draw the non-favorable outcome. For one stimulus per outcome combination, the good outcome could be obtained with a go or a nogo choice, respectively. Correct choices led to the more favorable outcome in 80% of the trials, while the non-favorable outcome was received in the other 20% of the trials.

Sequence and time course of stimulus presentation in the task version for observational learners were as similar to the active version as possible (Fig. 1b). As mentioned above, observational learners completed the task in a yoked design in which an observing subject was shown a previous “active” subject’s choices. Participants were explicitly informed about this and advised that the previous subject’s choices were illustrated on the screen by a hand that was displayed hovering over the circle if the previous subject had responded. All subjects were asked to pay close attention to the observed choices and ensuing feedback.

For both active and observational learners, each learning block was followed by a test block in which no feedback was provided for the subject’s decision to respond or not respond; otherwise test trials were identical to active participants’ learning trials. Importantly, both active and observational learners were explicitly instructed to decide between responding and not responding on each trial, and to optimize their performance based on the feedback that had been provided in the learning blocks.

In total, the task comprised four learning and four test blocks with 40 trials (10 per combination) each. Trial order was randomized within each block. Subjects could take short breaks between blocks and were informed about their current score at the end of each block. In order to keep the subjects motivated and to prevent negative scores especially early on in the task, the starting score was set to 400 points. Observing subjects were instructed that they would receive both the points won by the active subject as displayed after each learning block, and the points they themselves won in the test trials. Of note, subjects were also informed that their final scores would not translate to a financial reward after testing because they would receive standardized course credit for participation. Task completion took approximately 50 min. Stimulus presentation and timing was controlled by Presentation software (Version 17.2, Neurobehavioral Systems, Inc., Berkeley, CA, USA).

### Procedure

Subjects were informed that the study investigated active and observational outcome-based learning. After written informed consent had been obtained, demographic information was collected and participants completed the MWT-B. Subsequently, subjects were seated in front of a computer screen at a viewing distance of approximately 50 cm. Before the experimental task was started, on-screen instructions and five learning and five test trials for practice were presented to the subjects. Of note, these practice trials contained colored geometric shapes instead of fractal images as imperative stimuli, as they were intended to familiarize the subjects with sequence and time course of stimulus presentation in the task without inducing learning just yet. The entire test session took approximately 60 min.

### Statistical analyses

Mean IQ estimates were compared between active and observational learners by means of an independent samples t test. This was done to ensure that potential effects of learning condition could not be attributed to group differences in intellectual abilities. In order to check for outliers with regard to task performance, accuracy rates (i.e., the percentages of correct responses in test blocks) according to action (go/nogo) and outcome valence (win/loss) were checked for subjects with scores that were more than 2 standard deviations (SDs) below (or above) the sample mean in more than two conditions. No outliers were identified, so all data from all subject could be used for analysis.

Accuracy rates were then analyzed with a repeated-measures analysis of variance (ANOVA) with the between-subjects factor *learning condition* (active/observational) and the within-subjects factors *block* (1–4), *action* (go/nogo), and *outcome valence* (win/loss). Greenhouse–Geisser correction was applied when the assumption of sphericity was violated. Significant main effects of block were resolved by means of linear trend analysis. Interactions were resolved by subordinate ANOVAs or post-hoc paired-sample *t* tests where appropriate. Bonferroni correction was applied to account for multiple testing when necessary.

In case the ANOVA yielded no significant main effects or interactions of the factor learning condition, we planned to perform complementary Bayesian hypothesis testing in order to confirm that this factor did not improve the predictive adequacy of the statistical model. To this end, a Bayesian repeated-measures ANOVA with the between-subject factor *learning condition* (active/observational) and the within-subjects factors *block* (1–4), *action* (go/nogo), and *outcome valence* (win/loss) was performed using JASP (Version 0.9.2; JASP Team, 2017; Wagenmakers, Love et al., 2018a; Wagenmakers, Marsman et al., 2018b). In the Bayesian ANOVA, the null model was compared against all other statistical models, i.e., models containing the main effects for the factors learning condition, action, outcome valence, and block as well as models containing any combination of these effects or respective interaction effects. Bayes factors (BFs) for each model were computed as the ratio of the predictive adequacy (i.e., the change from prior to posterior odds based on the present data) of each statistical model and the null model. Thus, the higher the BF, the more the evidence is in favor of the respective statistical model (Wagenmakers, Love et al., 2018a; Wagenmakers, Marsman et al., 2018b). BFs were classified as suggested by Lee and Wagenmakers (2014) (adapted from Jeffreys, 1998; see also (Wagenmakers, Marsman et al., 2018b), with values between 1 and 3 indicating anecdotal, values between 3 and 10 indicating moderate, values between 10 and 30 indicating strong, values between 30 and 100 indicating very strong, and values larger than 100 indicating extreme evidence for a specific model against the null model. The priors were set to *p*(*m*) = 0.006 for all 167 conceivable models, thus reflecting a uniform distribution of prior model probabilities. Since the complex 4 × 2 × 2 × 2 design of the present study resulted in a very large number of models, we applied Bayesian model averaging in order to quantify how much the data supported the inclusion of each effect. This procedure yields the change from prior to posterior odds (BF_Inclusion_) for each effect, taking into account each candidate models’ conclusions (Wagenmakers, Marsman et al., 2018b).

### Results

Mean IQ scores did not differ between active learners (mean = 112.29, SD = 7.94) and yoked observers (mean = 114.78, SD = 12.15; *p* = 0.482).

#### Standard repeated-measures analysis of variance

**Fig. 2:**
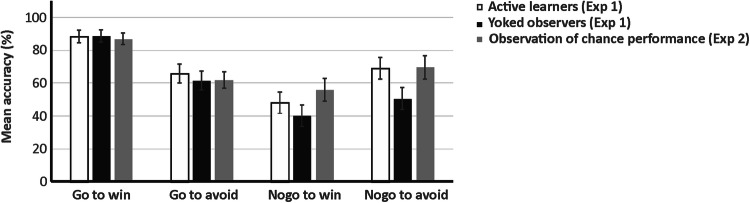
Mean performance accuracy according to action and outcome valence for active learners, yoked observers, and subjects who observed chance performance

Figure 2 shows mean performance accuracy according to action and outcome valence collapsed across blocks for active learners and yoked observers. The respective means according to block are provided as supplementary material. The ANOVA yielded a significant main effect of block (*F*_[2, 87]_ = 7.838, *p* < 0.001, *ƞ*_p_^2^ = 0.171). Linear trend analysis revealed that accuracy rates increased linearly across blocks (*F*_[1, 38]_ = 11.466, *p* = 0.002, *ƞ*_p_^2^ = 0.232). The main effect of action was also significant (*F*_[1, 38]_ = 26.172, *p* = 0.002, *ƞ*_p_^2^ = 0.232), with better performance on go (mean = 76.31% ± 2.69) compared to nogo trials (mean = 52.06% ± 3.89). These effects were further qualified by a significant block by action interaction (*F*_[2, 93]_ = 3.052, *p* = 0.042, *ƞ*_p_^2^ = 0.074). To resolve this interaction, separate univariate ANOVAs with the within-subjects factor block (1–4) were performed for go and nogo trials. For go trials, the main effect of block was significant (*F*_[3, 117]_ = 2.841, *p* = 0.042, *ƞ*_p_^2^ = 0.067) and did not reflect a linear (*p* = 0.221) but a cubic trend in accuracy rates across blocks (*F*_[1, 39]_ = 7.185, *p* = 0.011, *ƞ*_p_^2^ = 0.156). For nogo trials, the main effect of block was also significant (*F*_[2, 91]_ = 6.797, *p* < 0.001, *ƞ*_p_^2^ = 0.148) and reflected a linear increase in accuracy across blocks (*F*_[1, 39]_ = 11.683, *p* = 0.001, *ƞ*_p_^2^ = 0.231).

Furthermore, the action by outcome valence interaction was significant (*F*_[1, 38]_ = 37.396, *p* < 0.001, *ƞ*_p_^2^ = 0.496). Post-hoc paired-sample *t* tests revealed that performance accuracy was higher for go to win (mean = 88.81% ± 2.60) than for go to avoid losing (mean = 63.8% ± 4.01; *t*_39_ = 6.012, *p* < 0.001), and for nogo to avoid losing (mean = 59.88% ± 4.88) than for nogo to win (mean = 44.25% ± 4.59; *t*_39_ = -3.051, *p* = 0.004), thus confirming the asymmetric coupling of action and outcome valence.

Last, the block by outcome valence by learning condition interaction was significant (*F*_[2,91]_ = 5.450, *p* = 0.003, *ƞ*_p_^2^ = 0.125). To resolve this interaction subordinate ANOVAs with the within-subjects factors block (1–4) and outcome valence (win/loss) were performed separately for active and observational learners. For active learners, the main effect of block (*F*_[2,39]_ = 3.024, *p* = 0.059, *ƞ*_p_^2^ = 0.137 and the block by outcome valence interaction (*F*_[3, 57]_ = 2.597, *p* = 0.061, *ƞ*_p_^2^ = 0.120) merely approached significance. For yoked observational learners, the analysis yielded a significant main effect of block (*F*_[3,57]_ = 5.684, *p* = 0.002, *ƞ*_p_^2^ = 0.230), reflecting a linear increase in performance (*F*_[1,39]_ = 7.176, *p* = 0.015, *ƞ*_p_^2^ = 0.274), and a significant block by outcome valence interaction (*F*_[3,57]_ = 2.869, *p* = 0.044, *ƞ*_p_^2^ = 0.131). In order to resolve the interaction, separate univariate ANOVAs were performed for win and loss trials. The main effect of block was significant for win trials (*F*_[2,43]_ = 7.460, *p* = 0.001, *ƞ*_p_^2^ = 0.282), reflecting a linear increase in accuracy across blocks (*F*_[1,19]_ = 14.037, *p* < 0.001, *ƞ*_p_^2^ = 0.425), but not for loss trials (*p* = 0.127). These results indicate that a linear increase in accuracy over the course of the task was more pronounced in observational learners, particularly for win trials.

All other effects failed to reach significance (all *p* > 0.163).

#### Bayesian repeated-measures analysis of variance

Table 1 shows the results of the Bayesian analysis of effects. Note that this analysis averaged across all models that contained a specific factor (Bayesian model averaging): while the prior inclusion probability for a specific factor (P(incl)) is the summed prior probability of all models that include this factor, the posterior inclusion probability of a specific factor (P(incl|data)) is the summed posterior probability of all models that include this factor. The change from prior to posterior inclusion odds is expressed as BF_Inclusion_. The results show that the data strongly supported the inclusion of the main effects for the factors action and outcome valence, as well as the action by outcome valence interaction. Effects involving the factor learning condition received very weak support (all BFs_Inclusion_ < 1), as did all remaining effects.

**Table 1:** Results of the analysis of effects for data from active learners and yoked observers (Experiment 1)

Effects	P(incl)	P(incl|data)	BF_Inclusion_
Block	0.886	0.829	0.622
Action	0.886	1.000	> 10,000
Outcome valence	0.886	1.000	> 10,000
Learning condition	0.886	0.695	0.293
Block * action	0.503	0.120	0.135
Block * outcome valence	0.503	0.012	0.012
Block * learning condition	0.503	0.020	0.020
Action * outcome valence	0.503	1.000	> 10,000
Action * learning condition	0.503	0.467	0.864
Outcome valence * learning condition	0.503	0.245	0.321
Block * action * outcome valence	0.120	< 0.001	< 0.001
Block * action * learning condition	0.120	< 0.001	< 0.001
Block * outcome valence * learning condition	0.120	< 0.001	< 0.001
Action * outcome valence * learning condition	0.120	0.029	0.220
Block * action * outcome valence * learning condition	0.006	< 0.001	< 0.001

This analysis averaged across all models containing a specific factor. The prior inclusion probability for a specific factor (P(incl)) is the summed prior probability of all models that include this factor. The posterior inclusion probability of a specific factor (P(incl|data)) is the summed posterior probability of all models that include this factor. The change from prior to posterior inclusion odds is provided as BF_Inclusion_

### Discussion

In Experiment 1, individual subjects performed the orthogonalized go/nogo task either as active learners or based on observing a previous subject’s responses and subsequent feedback (yoked design) on the computer screen. In accordance with previous findings (Guitart-Masip, Economides et al., 2014a, b), results revealed a linear increase in performance irrespective of learning condition, indicating that active learners and observers were able to learn the stimulus-(non)response-outcome associations. Moreover, learning performance was generally better for go relative to nogo trials, which has also been observed in different variants of go/nogo tasks, including the orthogonalized version (Guitart-Masip et al., 2011; Guitart-Masip, Economides et al., 2014a, b; Ocklenburg et al., 2017), and may reflect generally increased task difficulty when response inhibition is required or could result from a general propensity to respond in experimental tasks.

Crucially, prior studies (Guitart-Masip et al., 2011; Guitart-Masip, Economides et al., 2014a, b) have yielded robust evidence for an asymmetric coupling of action and outcome valence in feedback-based learning. Learning to execute a response to obtain a reward (go to win) or to inhibit a response to avoid punishment (nogo to avoid losing) was easier than learning to inhibit a response to obtain a reward (nogo to win) or learning to execute a response to avoid losing (go to avoid) (Guitart-Masip, Duzel et al., 2014a, b). This result pattern was interpreted to reflect a conflict between Pavlovian control of behavior, which promotes active approach when rewards are anticipated, and inhibition or withdrawal when punishment is anticipated (Gray & MacNaughton, 2003), and the more flexible instrumental control that is driven by outcome valence. The present results replicate these findings.

Learning condition did not affect overall performance, which is consistent with findings from studies that applied other probabilistic learning tasks (Bellebaum et al., 2012; Bellebaum & Colosio, 2014; Rak et al., 2013). Importantly, the interaction of action and outcome valence was found to be comparable in active and observational learning, indicating that Pavlovian biases affected both learning types alike. Learning condition did, however, interact with outcome valence as a function of block: the linear increase in performance accuracy was more pronounced in observational learners, and particularly in the context of wins. However, Bayesian analysis of effects confirmed inclusion of the main effects for the factors action and outcome valence, and of the action by outcome valence interaction in the model, while providing very weak support for inclusion of the factors learning condition or block.

Taken together, the results from Experiment 1 appear to suggest that active and observational learning are similarly affected by Pavlovian biases, thus adding to evidence for similarities between processing of personal and vicarious rewards (Morelli et al., 2015). However, the possibility that observers merely imitated the responses of the active subjects rather than actually learned the stimulus-(non)response-outcome contingencies cannot be excluded. Therefore, a follow-up experiment (Experiment 2) was performed in which observational learners were presented with chance performance in order to test whether (a) their performance accuracy would still increase over the course of the task, thus reflecting true observational learning, and (b) a Pavlovian bias would still persist when chance performance was observed.

## Experiment 2

### Subjects

Twenty healthy adults (12 females, 8 males) were recruited by public advertisement at Heinrich-Heine-University Düsseldorf, Germany, or on social media. All had normal or corrected-to-normal vision, were naïve to the study’s intent, did not currently take any neurotropic medication, and had no history of neurological or psychiatric illnesses. Mean age was 22.7 years (SD = 2.0; age range 19 to 27 years). Mean IQ as determined with the MWT-B (Lehrl et al., 1995) was 112.90 (SD = 13.22) in this sample and did not differ from the sample tested in Experiment 1 (*p* = 0.834). Written informed consent was obtained from all participants prior to participation. As subjects were Psychology students, course credit was assigned for participation. The study conforms to the Declaration of Helsinki and received ethical clearance by the Ethics Board of the Faculty of Mathematics and Natural Sciences at Heinrich-Heine-University Düsseldorf, Germany.

### Experimental task and procedure

The experimental task and task instructions were identical to the ones used for observational learners in Experiment 1. However, the observational learners in the present sample actually observed one of four pseudorandomized trial sequences, all of which entailed chance performance (i.e., for each of the four combinations of action and outcome valence go to win, go to avoid, nogo to win, nogo to avoid, the same number of correct and incorrect responses were shown). Each of the four fractal images was assigned to each of the conditions in one of these sequences in order to prevent stimulus-specific learning effects. Testing procedures were otherwise identical to Experiment 1. Importantly, feedback probability for each decision was unchanged so that participants could still learn which choices led to what type of feedback.

### Statistical analyses

As done for Experiment 1, accuracy rates (i.e., the percentages of correct responses in test blocks) according to action (go/nogo) and outcome valence (win/loss) were first checked for outliers. There were no subjects with scores that deviated from the sample means by more than 2 SDs in more than two conditions. Hence, data from all subject were used for analysis. Accuracy rates were analyzed with a repeated-measures ANOVA with the within-subjects factors block (1–4), action (go/nogo), and outcome valence (win/loss). Greenhouse–Geisser correction was applied when the assumption of sphericity was violated. Linear trend analysis was performed in order to resolve the main effect of block. Interactions were resolved by post-hoc paired-sample *t* tests where appropriate. Bonferroni correction was applied to account for multiple testing when necessary. In addition, Bayesian hypothesis testing was used to determine if the learning condition had an impact on performance. To this end, data from the observational learners of Experiment 2 were analyzed together with data from active learners in Experiment 1, and a Bayesian repeated-measures ANOVA with the between-subjects factor *learning condition* (active/observational) and the within-subjects factors *block* (1–4), *action* (go/nogo), and *outcome valence* (win/loss) was performed as described above (see Experiment 1).

### Results

#### Standard repeated-measures analysis of variance (observational learners only)

Mean performance accuracy according action and outcome valence collapsed across blocks for learners observing chance performance is provided in Fig. 2. Respective descriptives according to the block factor are provided in the supplement. The ANOVA yielded a significant main effect of block (*F*_[3,57]_ = 4.020, *p* = 0.012, *ƞ*_p_^2^ = 0.175), reflecting a linear increase in performance over the course of the task (*F*_[1,19]_ = 13.484, *p* = 0.002, *ƞ*_p_^2^ = 0.415). Furthermore, the action by outcome valence interaction was significant (*F*_[1,19]_ = 16.870, *p* = 0.001, *ƞ*_p_^2^ = 0.470). Post-hoc paired-sample *t* tests showed that performance accuracy was higher for go to win (mean = 87.00% ± 3.53) than for go to avoid losing (mean = 62.00% ± 4.99; *t*_39_ = 4.660, *p* < 0.001). There was no difference between nogo to avoid losing (mean = 69.75% ± 7.12) and nogo to win (mean = 56.13% ± 7.01; *p* = 0.134). The main effect of action merely approached significance (*F*_[1,19]_ = 3.654, *p* = 0.071, *ƞ*_p_^2^ = 0.161), and all other effects were non-significant (all *p* > 0.157).

#### Bayesian repeated-measures analysis of variance (active and observational learners)

Table 2 shows the results of the analysis of effects for data from subjects who had observed chance performance and active learners from Experiment 1. Averaged across all candidate models, the data strongly supported the inclusion of the main effects for the factors action and outcome valence and the action by outcome valence interaction, while effects involving the factor learning condition received very weak support (all BFs_Inclusion_ < 1), as did all remaining effects. The overall result pattern was hence very consistent with results Experiment 1.

**Table 2:** Results of the analysis of effects for data from subjects who had observed chance performance and active learners from Experiment 1

Effects	P(incl)	P(incl|data)	BF _Inclusion_
Block	0.886	0.526	0.143
Action	0.886	1.000	> 10,000
Outcome valence	0.886	1.000	> 10,000
Learning condition	0.886	0.254	0.044
Block * action	0.503	0.047	0.048
Block * outcome valence	0.503	0.082	0.089
Block * learning condition	0.503	0.002	0.002
Action * outcome valence	0.503	1.000	> 10,000
Action * learning condition	0.503	0.074	0.079
Outcome valence * learning condition	0.503	0.047	0.049
Block * action * outcome valence	0.120	< 0.001	0.002
Block * action * learning condition	0.120	< 0.001	< 0.001
Block * outcome valence * learning condition	0.120	< 0.001	< 0.001
Action * outcome valence * learning condition	0.120	< 0.001	0.019
Block * action * outcome valence * learning condition	0.006	< 0.001	< 0.001

The prior inclusion probability for a specific factor (P(incl)) is the summed prior probability of all models that include this factor. The posterior inclusion probability of a specific factor (P(incl|data)) is the summed posterior probability of all models that include this factor. The change from prior to posterior inclusion odds is provided as BF_Inclusion_

### Discussion

The present data clearly show that observational learners did not merely imitate the responses they had seen, as their performance accuracy increased linearly over the course of the task and their performance levels were generally comparable to those in active learners and yoked observers in Experiment 1. The interaction between action and outcome valence was also observed, with better performance for go to win relative to go to avoid, while the difference between nogo to avoid and nogo to win was not significant. The latter result is in contrast to our findings in Experiment 1. It has to be noted, however, that results of previous studies with active learning are also inconsistent in this respect, because this difference has been found to be significant in one (Guitart-Masip, Economides et al., 2014a, b) and non-significant in another study (Guitart-Masip et al., 2011). It appears that Pavlovian interference with instrumental control may be more pronounced in the context of response execution than response inhibition, and our findings illustrate that this effect is not modulated by learning condition.

Bayesian analysis yielded result patterns identical to Experiment 1: The inclusion of the main effects for the factors action and outcome valence, and of the action by outcome interaction was very strongly supported, while all other effects received very weak support (all BFs_Inclusion_ < 1).

While Experiments 1 and 2 thus provide consistent evidence in favor of a Pavlovian bias in observational learning which is comparable to the one in active learning, both experiments did not comprise a manipulation check to determine if the observational learners had actually believed to be watching an active subject’s performance. Since there is a growing body of evidence that social contextual factors such as the presence of an uninvolved observer (Voegler et al., 2018), the representation of the task set of another individual during task sharing (Peterburs et al., 2019), or the degree of familiarity between subjects (Morelli et al., 2018) affect performance monitoring and reward processing for own and observed behavior, we decided to run a follow-up experiment that involved simultaneous testing of pairs of subjects, with one individual as the active and the other as the observational learner.

## Experiment 3

### Subjects

Forty-eight adult volunteers (27 females, 21 males) were recruited for participation at Heinrich-Heine-University Düsseldorf, Germany, by public advertisement and/or on social media. All had normal or corrected-to-normal vision and were naïve to the study’s intent. Mean age was 22.4 years (SD = 2.8; age range 18–30 years). None of the subjects had any history of neurological or psychiatric illnesses or was currently treated with neurotropic medication. Mean IQ as determined with the MWT-B (Lehrl et al., 1995) was 110.15 (SD = 10.21). Written informed consent was obtained from all participants prior to the experiment. Subjects received course credit for participation. The study conforms to the Declaration of Helsinki and received ethical clearance by the Ethics Board of the Faculty of Mathematics and Natural Sciences at Heinrich-Heine-University Düsseldorf, Germany.

### Experimental task

The experimental task was identical to the one used for active learners in Experiment 1. However, two subjects performed the task simultaneously, one as the active learner, and the other one as the observational learner. During learning blocks, the two subjects were seated next to each other, with the observing subject on the right. Responses by the active subject were recorded with an RB-844 USB response pad (Cedrus Corporation, San Pedro, CA, USA) to ensure that observers could easily identify button presses or the lack thereof. In this way the observer could learn by observing the actions and ensuing outcomes of the active person. At the end of each learning block, the active learner engaged in a block of test trials without feedback, but without being observed by the observer participant. The observing subject, instead, turned to the right by 90° to complete his/her own block of test trials on a separate computer with an identical RB-844 USB response pad. The computer screen was visually shielded from the active subject’s screen by means of a divider in order to prevent interference. After completion of the test block, the observing subjects moved back to the left to resume their position next to the active subject for the next learning block. In this way, learning could be assessed in both participants independently.

Subjects were informed that observers would receive both the points won by the active subject in the learning blocks and the points they themselves won in the test blocks, and that, accordingly, active subjects would receive the points they had won in the learning and the test blocks.

### Procedure

Subjects were informed that the study investigated active and observational outcome-based learning and that they, therefore, would be assigned the role of an active or an observational learner. After written informed consent had been obtained, demographic information was collected and subjects were positioned in front of a computer screen at a viewing distance of approximately 50 cm. Before the task was started, on-screen instructions were presented, and five learning and five test trials were completed in order to familiarize subjects with the task. Note that observing subjects watched active subjects during practice trials for learning blocks and actively completed practice trials for test blocks on the second computer. After finishing with the experimental task, participants completed the MWT-B. The entire test session took approximately 60 min.

### Statistical analyses

Statistical analyses were performed in accordance with the procedures described for Experiment 1. Outlier analysis did not identify any subjects whose accuracy scores deviated from the sample means by more than 2 SDs in more than two conditions.

### Results

Mean IQ scores did not differ (*p* = 0.072) between subjects serving as active (mean = 112.79, SD = 11.40) and observational learners (mean = 107.50, SD = 8.27).

#### Standard repeated-measures analysis of variance

**Fig. 3:**
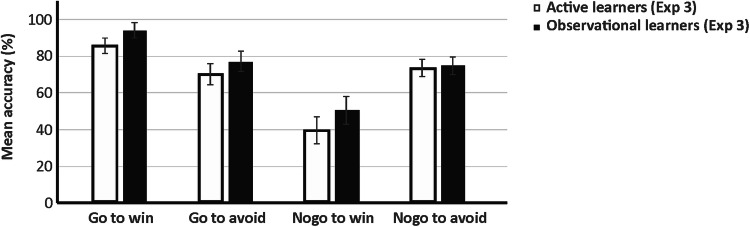
Mean performance accuracy according to action and outcome valence for active learners and observers who completed the task simultaneously (Experiment 3)

Figure 3 provides mean performance accuracy according to action and outcome valence for active and observational learners. Respective descriptives that include the block factor are provided in the supplement. The ANOVA yielded a significant main effect of block (*F*_[2, 177]_ = 12.722, *p* < 0.001, *ƞ*_p_^2^ = 0.217). Linear trend analysis showed that accuracy rates increased linearly across blocks (*F*_[1, 46]_ = 23.060, *p* = 0.002, *ƞ*_p_^2^ = 0.334). The main effect of action was also significant (*F*_[1, 46]_ = 39.824, *p* < 0.001, *ƞ*_p_^2^ = 0.464), with better performance on go (mean = 80.23% ± 2.55) compared to nogo trials (mean = 58.15% ± 3.39). The block by action interaction approached significance (*F*_[2, 104]_ = 2.750, *p* = 0.062, *ƞ*_p_^2^ = 0.056). These effects were further qualified by a significant block by action by learning condition interaction (*F*_[3, 138]_ = 4.365, *p* = 0.006, *ƞ*_p_^2^ = 0.087). In order to resolve this effect, subordinate ANOVAs with block and action as within-subjects factors were calculated separately for active and observational learners. The ANOVA for active learners yielded significant main effects of block (*F*_[2, 46]_ = 5.900, *p* = 0.005, *ƞ*_p_^2^ = 0.204), indicating a linear increase in performance throughout the task (*F*_[1, 23]_ = 10.278, *p* = 0.004, *ƞ*_p_^2^ = 0.309), and action (*F*_[1, 23]_ = 13.038, *p* = 0.001, *ƞ*_p_^2^ = 0.362), reflecting better performance in go (mean = 76.46% ± 4.18) relative to nogo trials (mean = 55.05% ± 5.56). The block by action interaction merely approached significance (*F*_[2, 49]_ = 2.934, *p* = 0.056, *ƞ*_p_^2^ = 0.113). For observational learners, analysis also revealed significant main effects of block (*F*_[3, 69]_ = 6.922, *p* < 0.001, *ƞ*_p_^2^ = 0.231), reflecting a linear increase in accuracy over the course of the task (*F*_[1, 23]_ = 12.837, *p* = 0.002, *ƞ*_p_^2^ = 0.358), and action (*F*_[1, 23]_ = 37.441, *p* < 0.001, *ƞ*_p_^2^ = 0.619), indicating better go (mean = 84.01% ± 2.91) than nogo performance (mean = 61.25% ± 3.87). In addition, the block by action interaction was significant (*F*_[2, 51]_ = 3.835, *p* = 0.024, *ƞ*_p_^2^ = 0.143). In order to resolve this interaction, linear trend analyses were performed to clarify the effect of block separately for go and nogo trials. A significant main effect of block (*F*_[2, 54]_ = 6.781, *p* = 0.001, *ƞ*_p_^2^ = 0.228), reflecting a linear performance increase (*F*_[1, 23]_ = 13.481, *p* = 0.001, *ƞ*_p_^2^ = 0.370) was only found for nogo but not for go trials (*p* = 0.730).

The main effect of outcome valence was also significant (*F*_[1, 46]_ = 4.483, *p* = 0.040, *ƞ*_p_^2^ = 0.089), indicating that accuracy was higher for loss (mean = 72.40% ± 2.60) than for win trials (mean = 65.99% ± 3.11). The block by outcome valence interaction approached significance (*F*_[3, 138]_ = 2.602, *p* = 0.055, *ƞ*_p_^2^ = 0.054). Crucially, the action by outcome valence interaction was significant (*F*_[1, 46]_ = 28.305, *p* < 0.001, *ƞ*_p_^2^ = 0.381). Post-hoc paired-sample *t* tests revealed that performance accuracy was higher for go to win (mean = 88.33% ± 2.92) than for go to avoid losing (mean = 72.14% ± 3.95; *t*_47_ = 3.482, *p* = 0.001), and for nogo to avoid losing (mean = 72.66% ± 3.38) than for nogo to win (mean = 43.65% ± 5.25; *t*_*47*_ = -5.118, *p* < 0.001), confirming the asymmetric coupling of action and outcome valence.

#### Bayesian repeated-measures analysis of variance

Table 3 shows the results of the analysis of effects for data from simultaneously performing subjects. Again, the results show that, averaged across all candidate models, the data strongly supported the inclusion of the main effects for the factors action and outcome valence, as well as the action by outcome valence interaction. Effects involving the factor learning condition received very weak support (all BFs_Inclusion_ < 1), as did all remaining effects. The overall result pattern was hence very consistent with the one obtained in Experiment 1.

**Table 3:** Results of the analysis of effects for data from simultaneously performing subjects (Experiment 3)

Effects	P(incl)	P(incl|data)	BF _Inclusion_
Block	0.886	0.944	2.176
Action	0.886	1.000	> 10,000
Outcome valence	0.886	1.000	> 10,000
Learning condition	0.886	0.383	0.080
Block * action	0.503	0.063	0.066
Block * outcome valence	0.503	0.032	0.033
Block * learning condition	0.503	0.006	0.006
Action * outcome valence	0.503	1.000	> 10,000
Action * learning condition	0.503	0.043	0.044
Outcome valence * learning condition	0.503	0.083	0.089
Block * action * outcome valence	0.120	< 0.001	< 0.001
Block * action * learning condition	0.120	< 0.001	< 0.001
Block * outcome valence * learning condition	0.120	< 0.001	< 0.001
Action * outcome valence * learning condition	0.120	0.003	0.019
Block * action * outcome valence * learning condition	0.006	< 0.001	< 0.001

The prior inclusion probability for a specific factor (P(incl)) is the summed prior probability of all models that include this factor. The posterior inclusion probability of a specific factor (P(incl|data)) is the summed posterior probability of all models that include this factor. The change from prior to posterior inclusion odds is provided as BF_Inclusion_

### Discussion

In Experiment 3, pairs of subjects were tested, with one individual as active and the other as observational learner, in order to create a more naturalistic setting. Results confirmed a significant main effect of action (better go than nogo performance) and the significant action by outcome valence interaction (better performance for go to win relative to go to avoid losing and for nogo to avoid losing relative to nogo to win). Comparing the result pattern to Experiment 1, effects of simultaneous testing of two subjects were rather subtle: in active subjects of simultaneously tested pairs, a gradual performance increase over the course of the task was found as well as overall better go than nogo performance. In observing subjects, aside from generally also better go than nogo performance, a linear increase in accuracy across blocks was only found for nogo but not go trials. This could be explained in terms of a ceiling effect, given generally high performance accuracy in go trials in observational learners, particularly in the go to win condition (see Fig. 3).

Bayesian analysis yielded comparable results to Experiment 1. Substantial predictive power was confirmed only for the main effects of the factors action and outcome valence, and their interaction.

## General discussion

The present study investigated the influence of action and outcome valence in active and observational feedback learning in order to determine if Pavlovian learning biases are specific to active task performance, or if they persist also under conditions of observational learning. In a series of three experiments, subjects completed an orthogonalized go/nogo task in which action (response execution or inhibition) and outcome valence (win or loss) were decoupled. In line with previous findings (Guitart-Masip, Huys et al., 2012a, b) and the a priori hypothesis, learning performance was modulated by both action and outcome valence as well as by the interaction of these factors. Importantly, and somewhat against our predictions, results revealed comparable Pavlovian learning biases in active and observational learning, with learning of go responses facilitated in the context of reward obtainment, and learning of nogo responses facilitated in the context of loss avoidance.

Pavlovian learning biases arise from a conflict between instrumental control of behavior, in which the behavioral output is entirely driven by outcome valence, and Pavlovian control, which favors approach or response execution in the prospect of reward and avoidance or response inhibition in the prospect of punishment (Guitart-Masip, Huys et al., 2012a, b). It has been suggested that Pavlovian control may represent evolutionary “hard-wired knowledge of good behavioral responses” that has proven advantageous despite its deleterious effect on learning in unusual environments (Guitart-Masip, Duzel et al., 2014a, b; Rangel et al., 2008). Along these lines, it seems plausible that Pavlovian biases should also be present in observational learning. On the other hand, previous research has provided evidence for reduced striatal recruitment for observational relative to active learning (e.g., Bellebaum et al., 2012; Kobza et al., 2012) and for processing of own relative to co-experienced/observed rewards (Morelli et al., 2018), likely reflecting reduced integration of outcome- and action-related information. This, in turn, could be associated with a reduction in Pavlovian learning biases. The present results clearly show that Pavlovian learning biases affect observational and active learning in a similar way, thus supporting the notion that these types of biases may be rather robust and deeply rooted. Experiment 2, in which participants observed chance performance and could thus learn similarly from correct and incorrect choices, clarified furthermore that the Pavlovian biases in observational learning do not reflect mere imitation of observed behavior, corroborating our previous finding that imitation plays only a minor role in observational learning (Bellebaum et al., 2016). The present study adds to evidence for similarities between active and observational learning and outcome processing that may be based on common mechanisms and shared neural substrates (Cooper et al., 2012; Morelli et al., 2015). Unfortunately, with its purely behavioral approach, the present study cannot directly inform about the underlying neural processes, but future imaging or electrophysiological studies might shed some light in this regard.

It has been suggested that active and observational learning may differ with regard to the type of knowledge representations they require (Kelly et al., 2003). Unfortunately, the present study is not suited to clarify whether observational learning may involve more explicit, declarative representations. Nicolle et al. (2011) asked subjects to provide explicit (subjective) estimates of the likelihood for winning for each of the stimuli in the learning task, a procedure that could be adopted in future studies to measure participants’ explicit knowledge about stimulus–action–outcome contingencies.

Interestingly, the present findings showed very similar result patterns for observation of virtual subjects and real subjects, indicating that an efficient observation manipulation does not require simultaneously performing subjects. While previous work has indicated that the degree of familiarity with the observed person (Morelli et al., 2018) and other inter-individual factors such as state or trait empathy (Thoma & Bellebaum, 2012) may modulate neural responses to observed rewards, these aspects were not manipulated in the present study. However, if Pavlovian biases are indeed rather hard-wired, it could be speculated that the asymmetric coupling between action and outcome valence might not be affected by these factors in observational learning. This could be addressed in future studies.

Another limitation of the present study relates to the fact that subjects did not receive performance-dependent payout. It could be speculated that receiving actual money in a performance-dependent manner might have increased motivation and thereby affected learning rates. Along these lines, future investigations should determine whether Pavlovian biases can be overcome more easily when subjects expect performance-dependent payout.

## Electronic supplementary material

Below is the link to the electronic supplementary material.

Supplementary file 1 (DOCX 284 kb)
