# Supplementary material for: Asymmetric coupling of action and outcome valence in active and observational feedback learning
Source: Psychol Res. 2020 Apr 22;85(4):1553–66. doi: 10.1007/s00426-020-01340-1 (PMC8211594; doi:10.1007/s00426-020-01340-1)

**SUPPLEMENTARY MATERIAL**

**Asymmetric coupling of action and outcome valence**

**in active and observational feedback learning**

Jutta Peterburs^1^, Alena Frieling^1^, Christian Bellebaum^1^

^1^Institute of Experimental Psychology, Department of Biological Psychology, Heinrich-Heine-University Düsseldorf, Universitätsstraße 1, 40225 Düsseldorf, Germany

*Corresponding author: Jutta.Peterburs@hhu.de*

The following figures provide a full breakdown of descriptives for mean performance accuracy based on the factors *block, action*, and *outcome valence* for active learners and yoked observers in Experiment 1, for subjects who observed chance performance in Experiment 2, and for active and observational learners who completed the task simultaneously (Experiment 3).

**ONLINE RESOURCE 1:** Mean performance accuracy according to block, action, and outcome valence for active learners (A), yoked observers (B), and subjects who observed chance performance (C)**.**


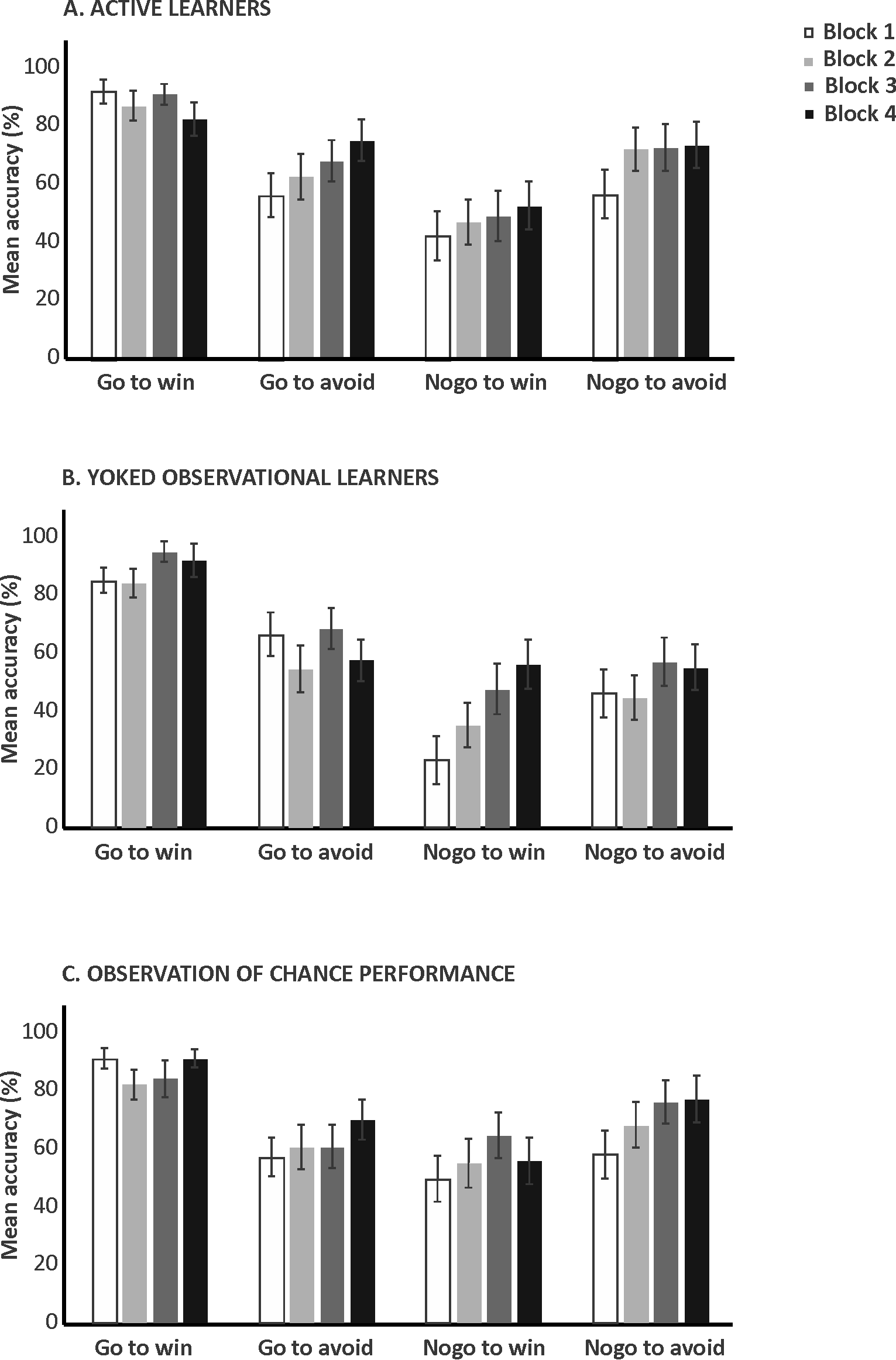


**ONLINE RESOURCE 2:** Mean performance accuracy according to block, action, and outcome valence for active learners (A) and observers (B) who completed the task simultaneously (Experiment 3)


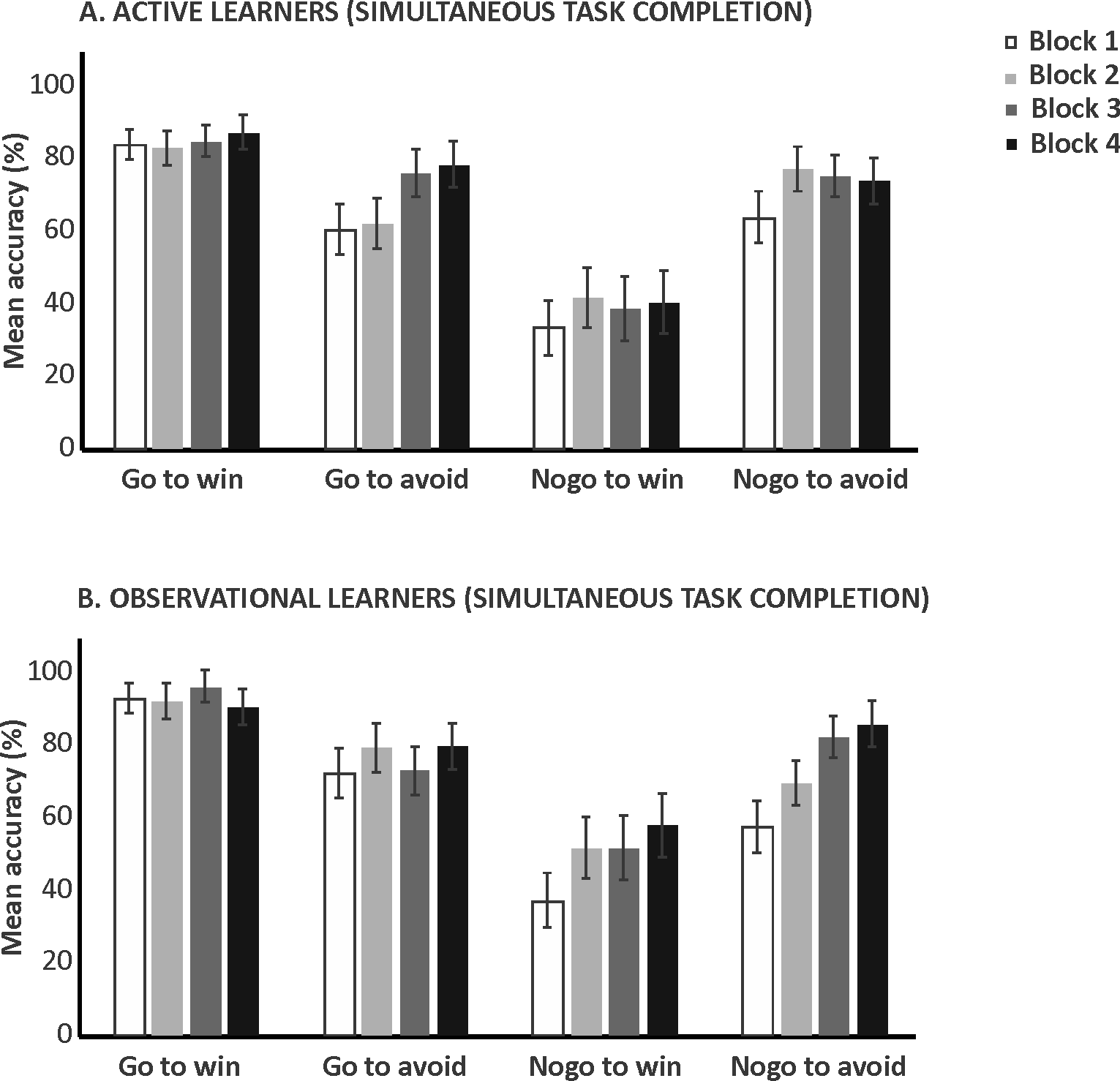

Supplement: Supplementary file 1 — (DOCX 284 kb) [file 426_2020_1340_MOESM1_ESM.docx]
